# Genome-Wide Characterization of the Sulfate Transporter Gene Family in Oilseed Crops: *Camelina sativa* and *Brassica napus*

**DOI:** 10.3390/plants12030628

**Published:** 2023-01-31

**Authors:** Parviz Heidari, Soosan Hasanzadeh, Sahar Faraji, Sezai Ercisli, Freddy Mora-Poblete

**Affiliations:** 1 Faculty of Agriculture, Shahrood University of Technology, Shahrood 3619995161, Iran; 2 Department of Plant Breeding, Faculty of Crop Sciences, Sari Agricultural Sciences and Natural Resources University (SANRU), Sari 4818168984, Iran; 3 Department of Horticulture, Faculty of Agriculture, Ataturk University, 25240 Erzurum, Turkey; 4 Institute of Biological Sciences, University of Talca, Talca 3460000, Chile

**Keywords:** bioinformatics, biotic stresses, regulatory mechanisms, protein structure, gene expression, evolutionary analysis

## Abstract

Sulfate transporters (SULTRs) are responsible for the uptake of sulfate (SO_4_^2−^) ions in the rhizosphere by roots and their distribution to plant organs. In this study, SULTR family members in the genomes of two oilseed crops (*Camelina sativa* and *Brassica napus*) were identified and characterized based on their sequence structures, duplication events, phylogenetic relationships, phosphorylation sites, and expression levels. In total, 36 and 45 putative *SULTR* genes were recognized in the genomes of *C. sativa* and *B. napus*, respectively. SULTR proteins were predicted to be basophilic proteins with low hydrophilicity in both studied species. According to the observed phylogenetic relationships, we divided the SULTRs into five groups, out of which the SULTR 3 group showed the highest variation. Additionally, several duplication events were observed between the *SULTRs*. The first duplication event occurred approximately five million years ago between three *SULTR 3.1* genes in *C. sativa.* Furthermore, two subunits were identified in the 3D structures of the SULTRs, which demonstrated that the active binding sites differed between *C. sativa* and *B. napus*. According to the available RNA-seq data, the *SULTRs* showed diverse expression levels in tissues and diverse responses to stimuli. *SULTR 3* was expressed in all tissues. *SULTR 3.1* was more upregulated in response to abiotic stresses in *C. sativa*, while *SULTR 3.3* and *SULTR 2.1* were upregulated in *B. napus*. Furthermore, *SULTR 3* and *SULTR 4.1* were upregulated in response to biotic stresses in *B. napus*. Additionally, the qPCR data showed that the *SULTRs* in *C. sativa* were involved in the plant’s response to salinity. Based on the distribution of cis-regulatory elements in the promoter region, we speculated that *SULTRs* might be controlled by phytohormones, such as ABA and MeJA. Therefore, it seems likely that *SULTR* genes in *C. sativa* have been more heavily influenced by evolutionary processes and have acquired further diversity. The results reveal new insights of the structures and functions of SULTRs in oilseed crops. However, further analyses, related to functional studies, are needed to uncover the role of SULTRs in the plants’ development and growth processes, as well as in their response to stimuli.

## 1. Introduction

Sulfur (S) is a macronutrient that is required for the biosynthesis of amino acids (such as cysteine (Cys) and methionine (Met)), vitamins, cofactors, and glutathione (GSH), as well as secondary metabolites; therefore, (S) is a vital element for plant growth, development, and stress response [1,2,3]. Root cells take up sulfate (SO_4_^2−^) in the form of S through a proton codependent process. The uptake and assimilation of sulfate resources that are available in the environment produce essential sulfur (S) metabolites that are crucial for development and stress responses, which is critical for plants and microbes [4]. The soil sulfate content can be modified by various factors, such as the dissimilation of soil microbes, the weathering of S-containing minerals, human activities that modify the deposition of S into the ecosystem, and climate change [1]. Therefore, the available sulfate content in soil can also be altered because of the ability of plant root systems to absorb nutrient compounds according to their requirements and material accessibility. It has been reported that in comparison to other micronutrients, sulfate only has a gentle and limited effect on root structures [5]. To meet demands of S required for the S-containing metabolite synthesis, plant membrane transport systems and their related metabolic enzymes optimize sulfate uptake, acquisition, storage, and use [6]. The uptake and distribution of sulfate in plants are facilitated by networks of sulfate transporters (SULTRs), which are encoded by a multigene family [7]. The H^+^/SO_4_^2−^ co-transporter SULTRs have been reported to contain 12 transmembrane domains, along with a carboxyl-terminal region, i.e., the so-called STAS (sulfate transporter/anti-sigma factor), which is suggested to play an important role in transporters’ activity and their interactions with other proteins [1,8].

The involvement of SULTRs in the transportation of S within plants was first reported by Smith et al. [9]. SULTRs are characterized by 12 transmembrane domains (TMDs) and an anti-sigma factor antagonist (STAS) domain at the C-terminus, which is critical for sulfate transporter activity [10]. The genomes of higher plants, such as *Arabidopsis thaliana*, rice, wheat, sorghum, and apple, have been reported to have 12, 12, 11, 10, and 9 *SULTR* genes, respectively [11,12,13,14]. The SULTR family has been well characterized in *Arabidopsis*, and sulfate transporters can be divided into four main groups based on their sequence resemblance, function, and location. The first group includes AtSULTR 1.1, AtSULTR 1.2, and AtSULTR 1.3, which are all high-affinity S transporters [15]. AtSULTRs 1.1 and 1.2 are co-localized in root hairs and epidermal and cortical cells in roots, and they are both responsible for the uptake of sulfate from soil [16,17]. Nevertheless, despite their common function, AtSULTR 1.1 predominantly operates under the conditions of S deficiency, while AtSULTR 1.2 operates efficiently under the conditions of either sulfur abundance or sulfur deficiency [18]. AtSULTR 1.3 is localized in the phloem, and cooperates in the source-sink distribution of sulfate [19]. The second group consists of two low-affinity transporters, AtSULTR 2.1 and AtSULTR 2.2, which are responsible for the transportation of sulfate from root to shoot [20]. The third group comprises five members (AtSULTR 3.1-5) and is the largest group. However, the precise functions of these members have not been fully established. It has been reported that SULTR 3.1, which transports sulfate to chloroplasts, could have a role in helping plants to withstand abiotic stresses [21]. Additionally, SULTR 3.5 has been reported to co-express with SULTR 2.1 to enhance the uptake of sulfate and facilitate its transportation from root to shoot under conditions of S deficiency [22,23]. The fourth group of transporters, SULTR 4.1 and SULTR 4.2, have been demonstrated to be tonoplast localized transporters that release sulfate from vacuoles into the cytosol [24,25]. As well as the study on *A. thaliana*, many other studies have been conducted to functionally characterize SULTRs in crops. For instance, 14 putative *SULTR* genes have been identified in rapeseed (*Brassica napus*), among which only those from group 1 and group 4 were induced in response to S deficiency [26]. In another study, 28 putative *SULTR* genes were identified in the soybean (*Glycine max*) genome and *GmSULTR 1.2b* was confirmed to have important roles in sulfate uptake and improving plants’ tolerance to sulfur deficiency [27]. In the potato (*Solanum tuberosum*) genome, 12 *SULTR* genes have been identified and the members of group 3 (StSULTR3s) were potentially proven to be involved in biotic/abiotic stress responses through MYB TFs, which play crucial roles in the modulation of StSULTR3s under these circumstances [28]. The maize (*Zea mays* L.) genome has been shown to include eight putative *SULTR* genes, all of which were induced by drought and heat stresses, except for *ZmSULTR 3.3* [29]. In addition, various studies have confirmed that *SULTRs* can be responsive to heavy metal exposure [30,31]. Despite the progress that has been made in the functional characterization of plant *SULTRs*, there are still more important crops that need to be investigated. *Camelina sativa* is an oilseed crop from the Brassicaceae family that has many qualities, including low inputs, great adaptation and resistance abilities, short life cycles, and easy genetic transformation, which have turned *C. sativa* into an ideal model plant [32,33]. Moreover, *C. sativa* is becoming more important as a biofuel [34,35]. Although oilseed plants typically have very high S demands [36], a study on the response of *C. sativa* to various fertilizers showed that the seed yields and oil contents of *camelina* seeds were not affected by sulfur fertilization [37]. In order to develop S-efficient crops and crop varieties that are tolerant to S deficiency, it is necessary to identify and characterize SULTRs, especially in low-input crops, such as *C. sativa*. To the best of our knowledge, there are no available reports on the genome-wide analysis of *SULTR* genes in *C. sativa*, except for one study that reported the upregulation of *SULTR 3.4* in *C. sativa* under salinity stress [38]. In this study, resources were employed to distinguish the regulation roles of *SULTR* genes in various cellular processes, especially in response to stimuli. *B. napus* is another well-known oilseed plant containing appreciable amounts of erucic acid. In the present study, we focused on SULTR sequences in the *C. sativa* and *B. napus* genomes, and compared and discussed their adjustments and their possible engagement in protection mechanisms against unfavorable environmental stimuli. We also highlighted the potential properties of these genes that could help to facilitate sulfate uptake.

## 2. Results

### 2.1. SULTR Properties in Camelina sativa and Brassica napus

**Figure 1:**
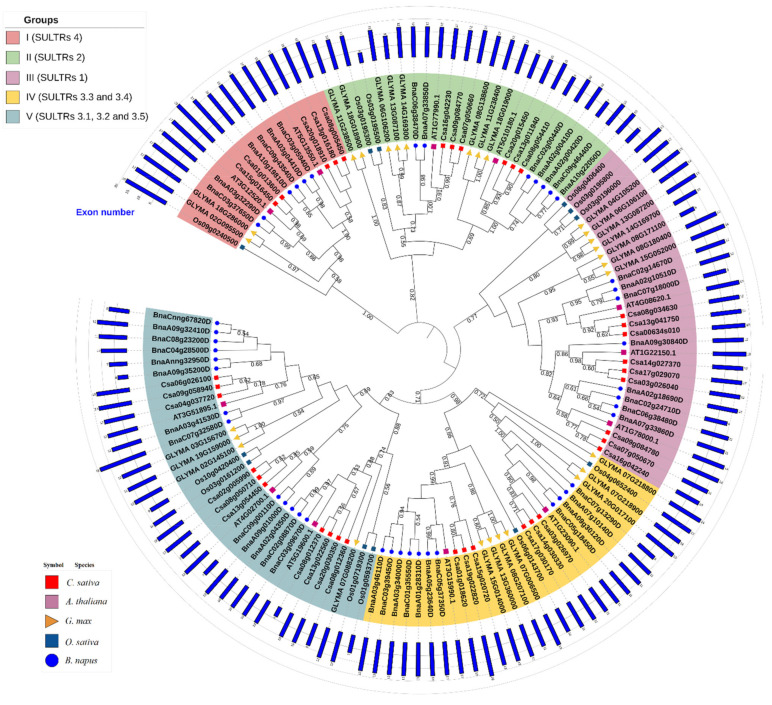
The phylogenetic tree of the SULTRs from *Camelina sativa, Brassica napus, Arabidopsis thaliana, Glycine max*, and *Oryza sativa*. The exon numbers for the SULTR coding genes are shown in the blue bar (more details related to the gene structures are provided in Table S1).

In the current study, 36 and 45 putative SULTR genes were recognized in the genomes of *C. sativa* and *B. napus*, respectively (Table S1). The SULTRs of the two oilseed crops were characterized and compared according to their coding DNA sequences (CDS) and protein lengths, exon numbers, isoelectric points (pIs), molecular weights (MWs), grand average of hydropathy (GRAVY) values, and instability indices (Table S1 and Table 1). Our results showed that the physicochemical properties of the SULTR proteins in the two studied plants were almost identical to each other. For instance, the MWs ranged from 29.07 to 91.99 kDa in *C. sativa*, and from 28.94 to 83.86 kDa in *B. napus*. Additionally, the pI values ranged from 7.41 to 9.93 in C. sativa, and from 7.11 to 10.71 in B. napus. Moreover, the GRAVY values varied from 0.271 to 0.624 in C. sativa, and from 0.108 to 0.621 in B. napus. Based on the instability indices, 83% and 73% of SULTR proteins were predicted to be stable proteins in *C. sativa* and *B. napus*, respectively. In addition, the exon numbers varied from 4 to 20 in *C. sativa* and from 4 to 19 in B. napus (Figure 1 and Table 1). Overall, the SULTR proteins were predicted to be basophilic proteins with low hydrophilicity.

### 2.2. Phylogenetic Analysis and Classification of the SULTR Gene Family

**Figure 2:**
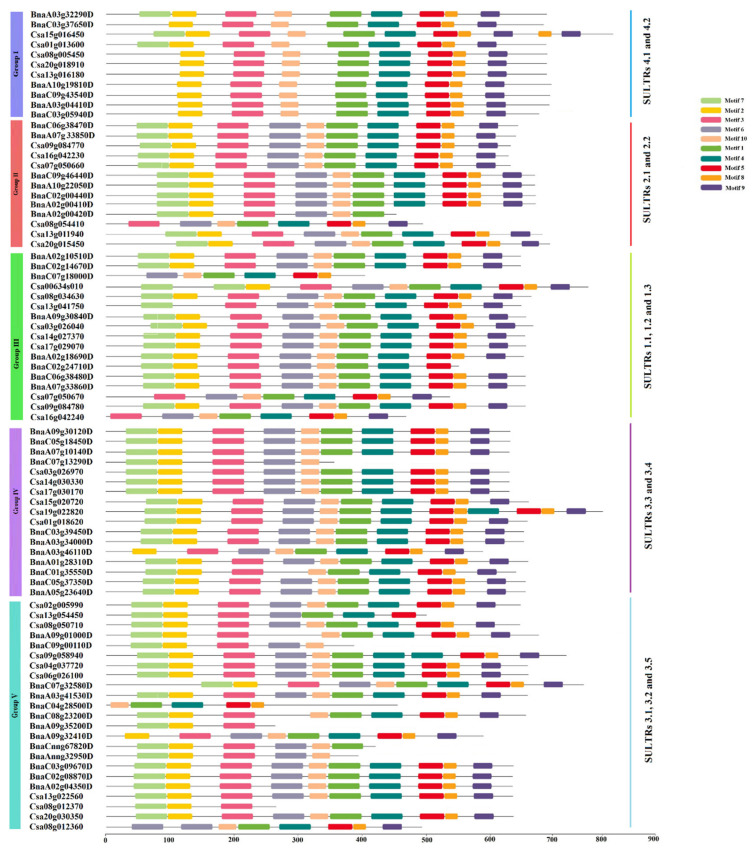
The distributions of the conserved motifs in the SULTRs from *Camelina sativa* and *Brassica napus*. The grouping was based on the phylogenetic tree. The sequences of the conserved motifs are presented in Figure S1.

In the present study, a phylogenetic tree of the SULTR proteins was created, including 45 SULTRs from *B. napus*, 36 SULTRs from *C. sativa*, 28 SULTRs from *Glycine max*, 12 SULTRs from *Oryza sativa*, and 12 SULTRs from *Arabidopsis thaliana* (Figure 1). The studied SULTRs were classified into five main groups: 16 SULTRs from SULTRs 4.1 and 4.2 were categorized into group 1; SULTRs 2.1 and 2.2 were clustered into group 2; 30 SULTRs from SULTRs 1.1, 1.2, and 1.3 were assigned to group 3; 28 proteins from SULTRs 3.3 and 3.4 were included in group 4; 34 SULTRs from SULTRs 3.1, 3.2, and 3.5 were located in group 5 (Figure 1). The SULTRs from monocot model plant (rice) were very different from the dicot samples. Moreover, the SULTRs from *C. sativa* and *B. napus* were evaluated and compared according to the conserved motifs. Overall, 10 conserved motifs were recognized in the protein sequences of the SULTRs, among which motif 6 was not observed in the SULTRs in group 1 (Figure 2). Additionally, 10 conserved motifs were identified in SULTRs 2.1 and 2.2, except the SULTR 2.1 from *C. sativa* only showed eight conserved motifs. Furthermore, SULTRs 1.1, 1.2, and 1.3 and 3.1, 3.2, and 3.5 were very diverse, according to the patterns of their motif distributions (Figure 2). Motifs 7 and 2 were frequently observed in the SULTR proteins and showed potential as screening markers for members of this family.

### 2.3. Evolutionary Processes in the MGT Genes of Citrullus lanatus and Cucumis sativus

**Figure 3:**
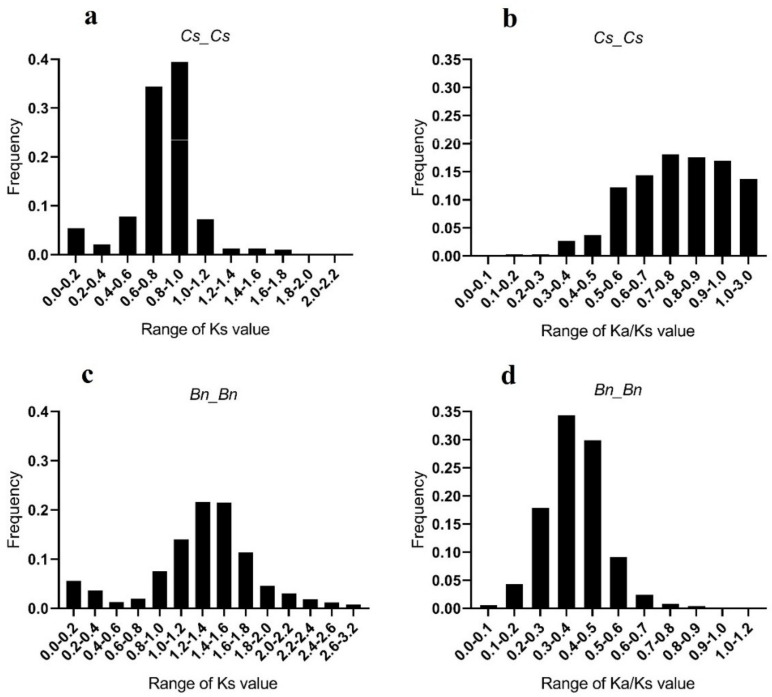
The frequency of Ks and Ka/Ks values in the *SULTRs*: (**a**) the frequency of Ks values in the *SULTRs* of *C. sativa* (Cs); (**b**) the frequency of the Ka/Ks values in the *SULTRs* of *C. sativa* (Cs); (**c**) the frequency of Ks values in the *SULTRs* of *Brassica napus* (Bn); (**d**) the frequency of the Ka/Ks values in the *SULTRs* of *Brassica napus* (Bn). The full details of the duplicated *SULTRs* are provided in Table S2.

**Figure 4:**
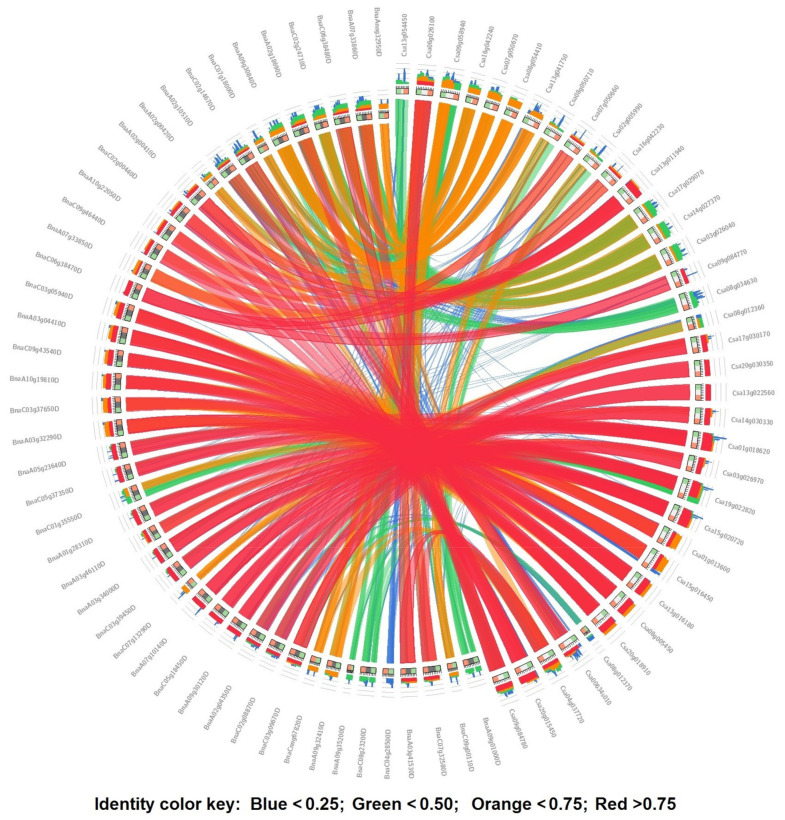
The synteny relationships between the *SULTRs* from *Camelina sativa* and *Brassica napus*.

In this study, to investigate the duplication events that have occurred in the SULTR gene family in *C. sativa* and *B. napus*, the synonymous (Ks), non-synonymous (Ka), and Ka/Ks values of each duplicated gene pair were calculated (Figure 3 and Table S2). The Ks values of the *SULTRs* in *C. sativa* were frequently between 0.6 and 1.0 (Figure 3a), while the Ka/Ks values were frequently between 0.7 and 0.9 (Figure 3b). In contrast, the Ks and Ka/Ks values of the *SULTRs* in the *B. napus* genome differed from those in *C. sativa*, with the Ks values frequently being between 1.2 and 1.6 (Figure 3c) and the Ka/Ks values frequently ranging from 0.3 to 0.5 (Figure 3d). In *C. sativa*, the first duplication event was predicted to have occurred around five million years ago (MYA) between three *SULTR 3.1 genes*, including *Csa06g026100-Csa04g037720* and *Csa09g058940-Csa04g037720*, while the first duplication event in *B. napus* occurred approximately three MYA between two *SULTR 3.1 genes*, *BnaA03g41530* and *BnaA09g35200* (Table S2). Several synteny blocks were observed between the *SULTRs* from *C. sativa* and *B. napus* (Figure S2). Additionally, three *SULTR* 1.3 genes (*Csa17g029070, Csa14g027370*, and *Csa03g026040*), four *SULTR* 3 genes (*Csa13g054450*, *Csa08g050710, Csa02g005990*, and *Csa08g012360*), and a *SULTR 1.1* gene (*Csa08g034630*) from *C. sativa* showed fewer synteny relationships with *SULTRs* from *B. napus* (Figure 4).

### 2.4. Transmembrane Structures of SULTRs

**Figure 5:**
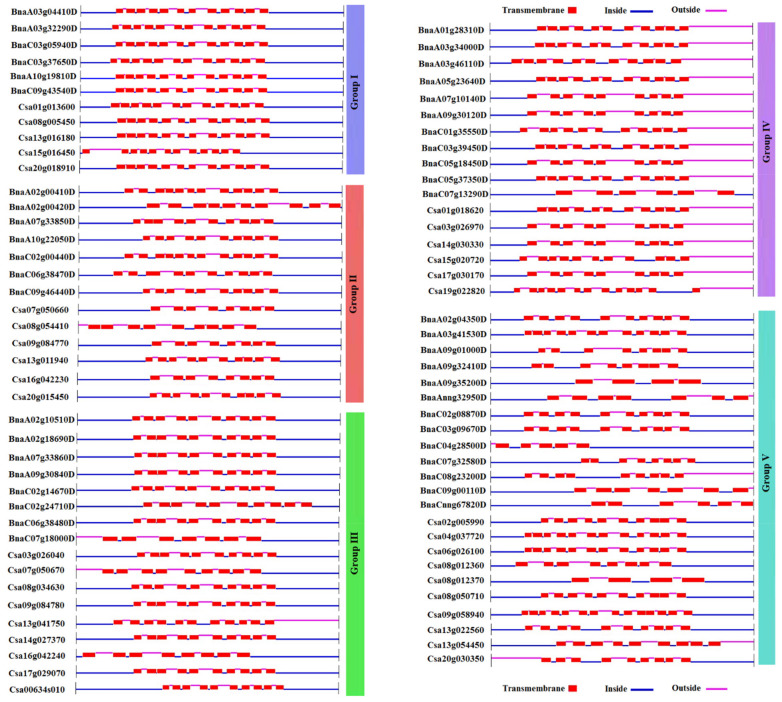
The transmembrane structures of the SULTRs in *C. sativa* and *B. napus*. The grouping was based on the phylogenetic tree.

The SULTR proteins from different groups were compared based on their transmembrane structures *in C. sativa* and *B. napus* (Figure 5). In group 1, 12 transmembrane helices and 11 pores were identified in all SULTRs. However, the SULTRs in *B. napus* showed similar structures based on the positions of the transmembrane helices while the structures in *C. sativa* were diverse. Additionally, the number of transmembrane helices in the group 2 SULTRs ranged from 10 to 12 in *B. napus* and from 8 to 10 in *C. sativa*. Most of the SULTRs in *B. napus* showed 10 transmembrane helices with nine pores (except for BnaC07g18000D with seven transmembrane helices), while the number of transmembrane helices in *C. sativa* varied between 8 and 11. In group 4, the number of transmembrane helices in the SULTRs of *B. napus* ranged from 6 to 11, while the number of transmembrane helices in *C. sativa* ranged from 9 and 13. The SULTRs in group 5 were very diverse in terms of their transmembrane structures, in which between 4 and 14 transmembrane helices were observed.

### 2.5. 3D Structure Analysis of SULTRs

**Figure 6:**
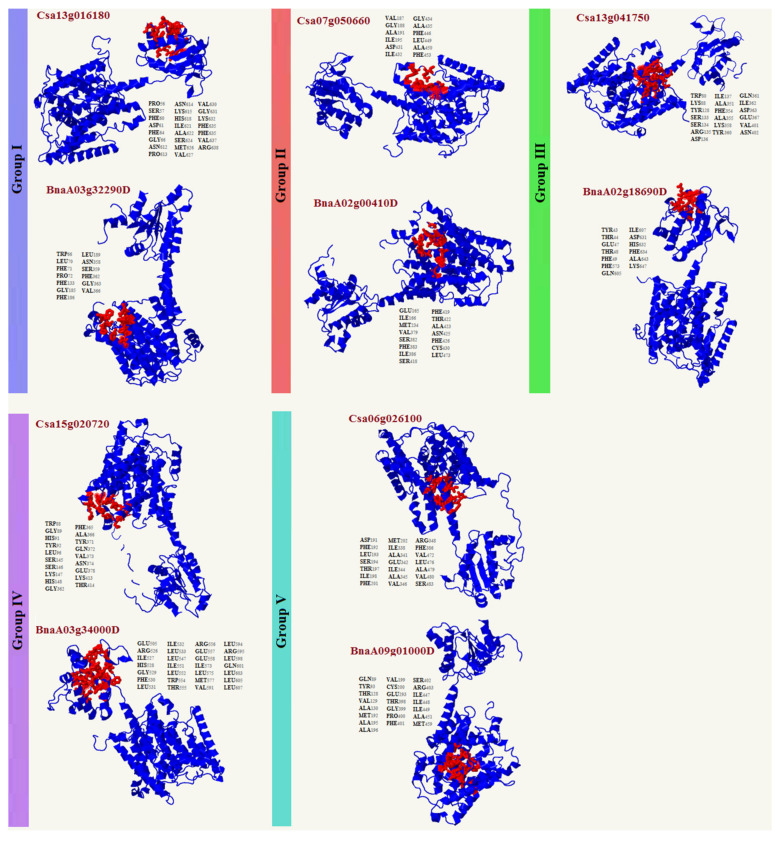
The three-dimensional docking analysis of the SULTRs in *C. sativa* and *B. napus*. The ligand binding sites are highlighted in red and lists of the binding sites are provided next to the protein structures.

Our analysis of the 3D structures revealed that the SULTRs in *C. sativa* and *B. napus* had two domains and that the active binding sites could be located in small or large subunits (Figure 6). These results showed that the SULTRs in *C. sativa* were different from those in *B. napus* (Figure 6). In the group 1 SULTRs, the valine (VAL), proline (PRO), phenylalanine (PHE), asparagine (ASN), lysine (LYS), glycine (GLY), and serine (SER) amino acids were frequently observed in the binding sites of SULTRs from *C. sativa*, while PHE, GLY, and leucine (LEU) were frequently observed in the binding sites of SULTRs from *B. napus* (Figure 6). In the group 2 SULTRs, PHE, GLY, and alanine (ALA) were more frequently observed in the binding sites of *C. sativa*, while PHE, SER, and isoleucine (ILE) were frequently observed in the binding sites of *B. napus*. Additionally, six amino acids, including SER, aspartic acid (ASP), LYS, ILE, ALA, and tyrosine (TYR), were more frequently observed in the binding sites of group 3 SULTRs in *C. sativa*, while PHE and threonine (THR) were frequently observed in the binding sites of *B. napus*. In the group 4 SULTRs, SER, GLY, histidine (HIS), and TYR were more commonly identified in the binding sites in *C. sativa*, while LEU, ILE, glutamate (GLU), and arginine (ARG) were frequently observed in the binding sites of *B. napus.* In the group 5 SULTRs, SER, PHE, ILE, ALA, VAL, LEU, and TYR were more frequently observed in the binding sites in *C. sativa*, while ALA, ILE, methionine (MET), VAL, and THR were frequently observed in the binding sites of *B. napus*.

### 2.6. SULTR Expression Analysis

**Figure 7:**
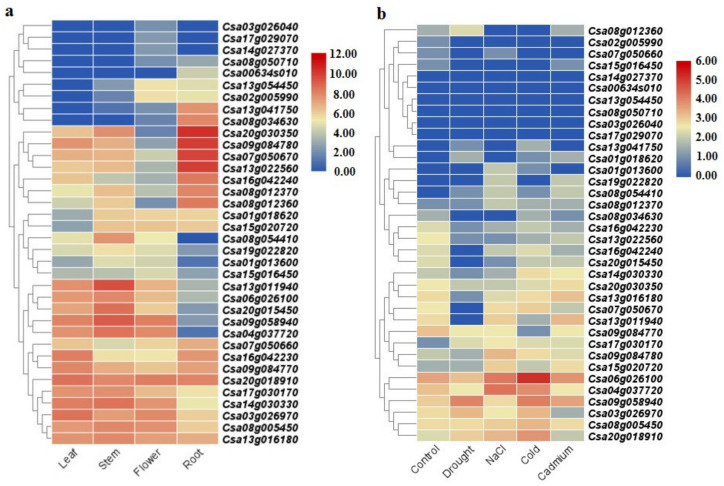
The expression levels of the *SULTRs* in *C. sativa*, based on the available RNA-seq data: (**a**) in different tissues; (**b**) in response to abiotic stresses.

**Figure 8:**
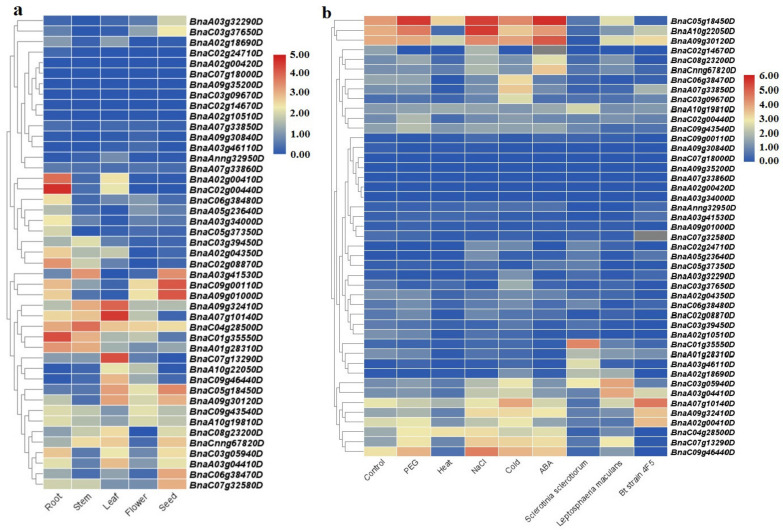
The expression levels of the *SULTRs* in *B. napus*, based on the available RNA-seq data: (**a**) in different tissues; (**b**) in response to abiotic and biotic stresses.

In this study, the expression patterns of *SULTRs* in *C. sativa* and *B. napus* were evaluated in different tissues and in response to stress (Figure 7 and Figure 8). We found that two *SULTR* 3.5 genes (*Csa20g030350* and *Csa13g022560*) and two *SULTR* 1.2 genes (*Csa09g084780* and *Csa07g050670*) were expressed more in the roots of *C. sativa*, while three *SULTR* 3.1 genes (*Csa06g026100*, *Csa09g58940*, and *Csa04g0377720*) and three *SULTR 2.1* genes (*Csa13g011940*, *Csa08g054410*, and *Csa20g015450*) were highly expressed in stem tissues (Figure 7a). In the leaf tissues of *C. sativa*, three *SULTR* 3.3 genes (*Csa17g030170*, *Csa14g030330*, and *Csa03g026970*), two *SULTR* 2.2 genes (*Csa16g042230* and *Csa09g084770*), and a *SULTR 4.1* gene (*Csa20g018910*) were highly expressed (Figure 7a). In response to abiotic stresses, *SULTR 3.1* was induced in *C. sativa* (Figure 7b). For example, *Csa06g026100* and *Csa04g037720* were expressed more in response to cold and salt stresses, while *Csa09g058940* was expressed more in response to drought, cold, and cadmium stresses. In addition, *Csa20g018910* (which is a chloroplast *SULTR 4.1*) was expressed more under cold stress (Figure 7b). Additionally, the *SULTRs* of *B. napus* showed diverse expression levels in tissues and in response to abiotic and biotic stresses (Figure 8). We found that two *SULTR 2.1* genes (*BnaA02g00410D* and *BnaC02g00440D*), a *SULTR 3.4* gene (*BnaC01g35550D*), and a *SULTR 3.5* gene (*BnaC02g08870D*) were highly expressed in the root tissues of *B. napus*, while two *SULTR 3.2* genes (*BnaC09g00110D* and *BnaA09g01000D*), two *SULTR 3.1* genes (*BnaA03g41530D* and *BnaC07g32580D*), a *SULTR 3.3* gene (*BnaC05g18450D*), and a *SULTR 2.2* gene (*BnaC06g38470D*) were expressed in seeds (Figure 8a). In the stem tissues of *B. napus*, two *SULTR* 3 genes (*BnaA03g41530D* and *BnaC04g28500D*) were highly expressed, while three *SULTR* 3 genes (*BnaA09g32410D*, *BnaA07g10140D*, and *BnaC07g13290D*), a *SULTR 2.1* gene (*BnaC09g46440D*), and a *SULTR 4.1* gene (*BnaA03g04410D*) were expressed in leaf tissues (Figure 8a). Furthermore, two *SULTR 3.3* genes (*BnaC05g18450D* and *BnaA09g30120*) and two *SULTR 2.1* genes (*BnaA10g22050D* and *BnaC09g46440D*) were more upregulated in response to PEG, NaCl, and ABA treatment (Figure 8b). Interestingly, two *SULTR 2.1* genes (*BnaC06g38470D* and *BnaA07g33850D*) were differentially expressed in response to cold stress in *B. napus*. However, *BnaA07g10140D* (which is a *SULTR 3.3*) and *BnaC09g46440D* (which is a *SULTR 2.1*) were also upregulated under cold stress. In response to biotic stresses, two *SULTR 4.1* genes (*BnaC03g05940D* and *BnaA03g04410D*) were upregulated in response to the fungal pathogen *Leptosphaeria maculans*. In addition, a *SULTR 3.4* gene (*BnaC01g3550D*) and a *SULTR 3.3* gene (*BnaA07g10140D*) were expressed more in response to *Sclerotinia sclerotiorum* and *Bacillus thuringiensis* strain 4f5, respectively (Figure 8b).

### 2.7. SULTR Phosphorylation Prediction

**Figure 9:**
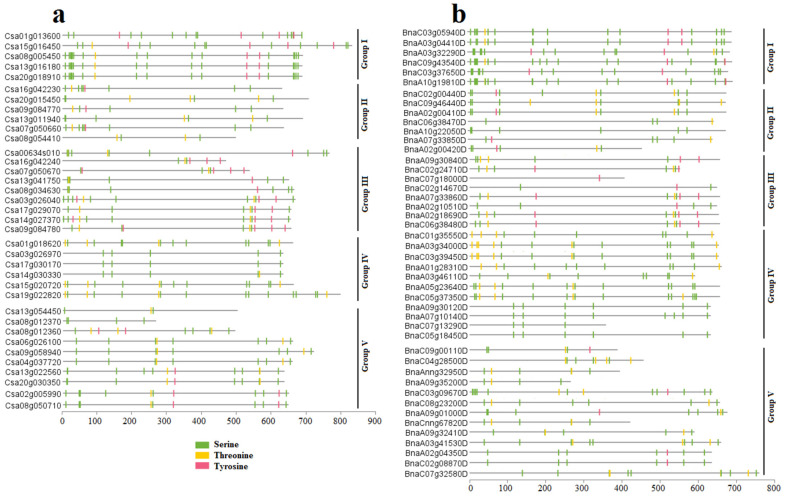
The prediction of phosphorylation sites in the SULTRs with scores ≥ 0.90 using the NetPhos 3.1 server: (**a**) *C. sativa*; (**b**) *B. napus*. The grouping was based on the phylogenetic tree.

The potential phosphorylation sites of the SULTRs in *C. sativa* and *B. napus* were predicted based on serine, threonine, and tyrosine amino acids (Figure 9). The potential phosphorylation sites in the SULTRs ranged from 3 (in Csa13g054450, which is a SULTR 3.2) to 21 (in Csa08g005450, which is a SULTR 4.1 from group 1), with an average of 10.28 sites per protein in *C. sativa* (Figure 9a). Interestingly, SULTR 4.1 showed a high potential for phosphorylation events in *C. sativa*. Additionally, the potential phosphorylation sites in the SULTRs in *B. napus* ranged from a site in BnaC07g18000D (which is a SULTR 1.1) to 23 sites in BnaA10g19810D (which is a SULTR 4.1), with an average of 9.71 sites per protein (Figure 9b). In addition, more phosphorylation sites were predicted in SULTR 4.1 in *B. napus*.

### 2.8. Distribution of Cis-Regulatory Elements in Promoter Sites

**Figure 10:**
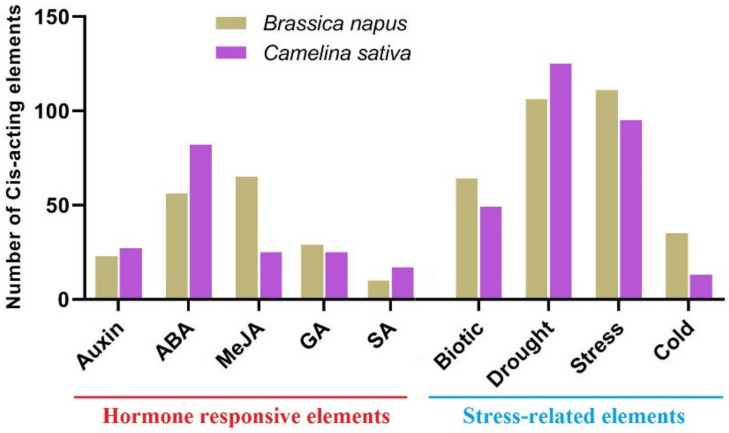
A comparison between the *SULTRs* from *C. sativa* and *B. napus* based on the number of cis-regulatory elements related to hormone and stress responses in promoter sites. More details are provided in Figures S3 and S4.

In this study, the distribution of cis-regulatory elements in the promoter sites of the *SULTRs* in *C. sativa* and *B. napus* was investigated (Figure 10, Figure S3, and S4). The *SULTRs* in *C. sativa* and *B. napus* were compared based on the cis-regulatory elements that were related to their responses to stress and hormones (Figure 10). The cis-regulatory elements associated with auxin, ABA, MeJA, GA, and SA responses were observed in the promoter regions of the *SULTRs*. The results revealed that the cis-regulatory elements of the ABA response were frequently distributed in the *SULTRs* from *C. sativa*, while the MeJA response elements were more commonly observed in *B. napus* (Figure 10). Additionally, the cis-regulatory elements related to biotic and cold stresses were more frequently observed in the *SULTRs* from *B. napus*, while those related to drought stress were more commonly observed in the promoter sites of the *SULTRs* from *C. sativa*.

### 2.9. Expression Patterns of SULTRs in Camelina in Response to Salinity Stress

**Figure 11:**
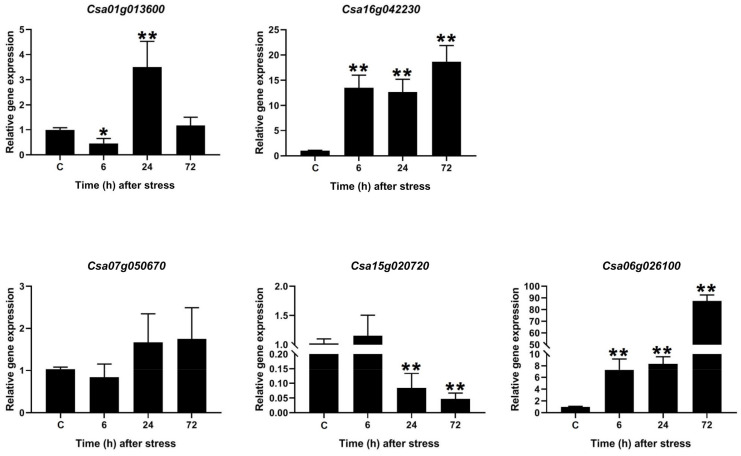
The expression levels of the *SULTRs* in *C. sativa* in response to salinity stress (i.e., 200 mM of NaCl) at three timepoints (6, 24, and 72 h after salt stress) and under control conditions (C, i.e., irrigation without NaCl), based on the qPCR data. Note: * and ** indicate significant differences between the expression levels following the salt treatment and those under normal conditions (based on a Student’s t-test) at *p* < 0.05 and *p* < 0.01, respectively.

To understand the potential roles of the *SULTR* genes in camelina plants, the expression levels of five selected genes were analyzed in response to salt stress (i.e., 200 mM of NaCl). The camelina *SULTR* genes illustrated different expression patterns under salinity (Figure 11). For instance, *Csa01g013600* (which is a SULTR 4.2) was downregulated after 6 h of salinity stress, while its expression was upregulated after 24 h. Moreover, *Csa16g042230* (which is a SULTR 2.2) and *Csa06g026100* (which is a SULTR 3.1) had similar expression patterns. Both genes were upregulated in response to salt stress and the maximum expression was observed after 72 h. In contrast, *Csa07g050670* (which is a SULTR 1.2) was not induced by salinity stress. The expression levels of one SULTR 3.4 gene (*Csa15g020720*) were significantly reduced after 24 h and 72 h of salt stress. Overall, these data showed that some SULTR family members were involved in the response to salt stress.

## 3. Discussion

The uptake and distribution of sulfate in plants are facilitated by networks of sulfate transporters, which are encoded by a multigene family (SULTRs) [7]. Due to the important role of sulfate in plants, the SULTRs in several plant species have been studied. For instance, the genomes of higher plants, such as *Arabidopsis thaliana*, rice (12 *SULTRs*), wheat (11 *SULTRs*), sorghum (10 *SULTRs*), and apple (9 *SULTRs*), have been identified [11,12,13,14]. In this study, we identified and characterized 36 and 45 putative *SULTR* genes in the genomes of *C. sativa* and *B. napus*, respectively (Table S1). More members of this gene family could be associated with changes in ploidy levels and genome sizes in *C. sativa* and *B. napus*, as well as duplication events in evolutionary processes [35,39]. Our investigations revealed that the SULTR proteins in the two studied plants had the same ranges for their physicochemical properties, i.e., MWs, pIs, GRAVY values, and instability indices. In addition, the exon numbers ranged from 4 to 20 in *C. sativa* and from 4 to 19 in *B. napus*. The similarities in their gene structures could indicate that significant evolutionary events have occurred in the plant genomes [40,41]. Our findings also suggested that the exon/intron patterns could provide new insights into the evolutionary relationships among the members of the gene family and that they could have originated from a common ancestor. Moreover, it has been reported that the exon number can affect expression levels, and that genes with lower exon numbers can be expressed quickly in response to environmental stresses [42,43]. SULTRs have been divided into four main classes based on their locations and functions [4]. In this study, the different SULTR classes were further separated based on our phylogenetic analysis. The SULTR 4 genes were very distinct from the other classes, while the SULTR 3 members varied significantly (Figure 1). Differences have also been observed between the SULTRs in the model monocot plant, rice, and dicot plants, indicating that the diversity in the SULTR gene family has occurred after the divergence of monocots and dicots [44,45]. According to our results for the conserved motifs in the SULTRs, some conserved sites were common between SULTR groups, which could be used to distinguish between specific groups.

According to our phylogenetic results, the camellia SULTRs were similar to the SULTRs of *B. napus*, although their evolutionary trends were different. Based on the Ka/Ks indices, the first duplication events in the SULTR genes in *C. sativa* occurred about five million years ago, while those in *B. napus* occurred three million years ago. Furthermore, it seemed that other members of the SULTR gene family originated from SULTR 3. Additionally, the Ka/Ks values revealed that the duplicated SULTRs in *B. napus* occurred under purifying (negative) selection, while both adaptive (positive) selection and purifying selection were observed in the *SULTRs* of *C. sativa* [46]. This suggested that the duplicated genes with conserved functions, pseudogenization, or both were possibly produced via purifying selection [47]. Interestingly, the results of our comparative synteny analysis revealed that several *SULTRs* from *C. sativa*, including three *SULTR 1.3* genes (*Csa17g029070*, *Csa14g027370*, and *Csa03g026040*), four *SULTR 3* genes (*Csa13g054450*, *Csa08g050710*, *Csa02g005990*, and *Csa08g012360*), and a *SULTR 1.1* gene (*Csa08g034630*), had fewer synteny relationships with the *SULTRs* from *B. napus* (Figure 4). It seemed that these genes could have been specifically developed during the evolution of the camellia, although more research is needed to determine their functions.

SULTRs can be classified into four groups based on their sequence structures, locations, and functions [48]. For instance, the genes in group 1 and group 2 are expressed more in root cells and vacuolar tissues, respectively [48,49]. In this study, the *SULTRs* in *C. sativa* and *B. napus* showed diverse expression levels in different tissues and in response to stresses. In the roots of *C. sativa*, two *SULTR 1.2* genes and two *SULTR 3.5* genes were expressed more, while two *SULTR 2.1* genes (*SULTR 3.4*, and *SULTR 3.5)* were highly expressed in the root tissues of *B. napus*. In the shoot tissues, *SULTRs 2, 3*, and 4 were expressed more. Interestingly, *SULTR* 3 showed a diverse range of functions and was expressed in all tissues, indicating that the members of this class were not specific to a tissue or organ. In addition, the members of *SULTR 3* varied greatly in terms of their transmembrane structure. Moreover, different expression patterns were observed between the members of the SULTR gene family in *B. napus* and camellia in response to stimuli. The *SULTR 3.1* genes were expressed more in response to abiotic stresses in *C. sativa*, while the *SULTR 3.3* and *SULTR 2.1* genes were more upregulated in *B. napus*. Several members of SULTR 3 play multiple roles and interact with abscisic acid (ABA) metabolism [21,22,23]. In the present study, SULTR 3 and SULTR 4.1 were upregulated in response to biotic stresses in *B. napus*, including bacterial and fungal pathogens. Additionally, the cis-regulatory elements related to ABA and MeJA responses were frequently observed in the promoter sites of the SULTRs. We concluded that the SULTRs could be controlled by phytohormones, especially the hormones related to stress, such as ABA and MeJA. These interactions could effectively induce the expression of the members of this gene family in response to stress. It can also be stated that the expression levels of different *SULTRs* could be correlated with hormone and stress response elements observed in the promoter regions. Additionally, the real-time PCR data revealed that the *SULTRs* in *C. sativa* had diverse expression patterns and were involved in the response to salt stress. This indicates that SULTRs could possibly interact with some transcription factors, such as MYB, and be indirectly involved in responses to abiotic stresses [28]. The prediction of the 3D structures revealed two subunits in the SULTRs and that the active binding sites differed between the subgroups (Figure 6). PHE, ALA, ILE, and VAL were identified as key amino acids in the binding sites, playing critical roles in the function and regulation of the SULTRs. Post-translational phosphorylation modifications can affect the function and possible interaction of proteins [50,51]. The prediction of the phosphorylation sites in the SULTRs revealed that the SULTR 4.1 genes had a high potential for influencing post-translation modifications, such as phosphorylation. The SULTR 4.1 and SULTR 4.2 genes have been reported to be tonoplast transporters, which allow sulfate to leave vacuoles to reach cytosol [24,25]. It seems that phosphorylation modifications play key roles in the activity of these transporters.

## 4. Materials and Methods

### 4.1. Identification of SULTR Genes in C. sativa and B. napus

To identify all sequences related to the SULTR family, the amino acid sequences of two conserved domains, including Sulfate_transp (PF00916) and STAS (PF01740), were used as queries in a BLASTP search of Ensembl Plants (https://plants.ensembl.org/index.html, accessed: 20 September 2022) in the protein databases of *C. sativa* and *B. napus*. Furthermore, orthologue genes were identified by following the same procedure for *Arabidopsis thaliana*, *Oryza sativa*, and *Glycine max*. All collected sequences were checked using the NCBI Conserved Domain Database (CDD) [52] and the Pfam database [53] to confirm the presence of domains related to the SULTRs [54]. The physiochemical properties, including molecular weight (MW), instability index, isoelectric point (pI), and GRAVY value, of the SULTRs were predicted using the ProtParam tool [55]. The TMHMM version 2.0 server was used to predict the transmembrane structures of the SULTRs in *C. sativa* and *B. napus* [56]. 

### 4.2. Phylogenetic and Conserved Motif Analyses

The amino acid sequences of all the identified *SULTRs* from five plant species, i.e., *C. sativa*, *B. napus*, *A. thaliana*, *O. sativa*, and *G. max*, were aligned using the online tool Clustal-Omega [57]. The entire phylogenetic relationships were constructed using the maximum likelihood (ML) method with 1000 bootstrap replicates using the IQ-TREE server [58]. Finally, a phylogenetic tree was created using the interactive tree of life tool (iTOL version 5) [59]. The conserved protein motifs in the SULTRs in *C. sativa* and *B. napus* were identified using the Multiple Expectation Maximization for Motif Elicitation program (MEME version 5.0.5) [60].

### 4.3. Promoter Analysis

In this study, 1500 bp upstream of the start codon in the *SULTRs* was selected as the promoter site, and these regions in *C. sativa* and *B. napus* were downloaded from Ensembl Plants. The sequence of each promoter site was screened to identify the conserved cis-regulatory elements using the PlantCARE tool [61]. Then, the cis-regulatory elements were classified based on their functions.

### 4.4. Ka/Ks Ratio and Duplication Analysis

In the present study, pairs of *SULTR* genes from each species (*C. sativa* and *B. napus*) with similarities of more than 85% were considered to be duplicated genes [62]. Additionally, the synonymous (Ks) and non-synonymous (Ka) indices were calculated for all gene pairs using the MEGAX software [63]. The time of divergence of the duplicated *SULTR* genes was estimated using the following equation: T = (Ks/2λ) × 10^−6^. (λ = 6.5 × 10^−9^) [64]. In addition, the synteny relationships between the SULTRs in each species, and between the orthologous genes of *C. sativa* and *B. napus*, were drawn using the Circos tool [65].

### 4.5. Gene Expression Analysis

In this study, the available RNA-seq data for *C. sativa* and *B. napus* were screened to extract the expression levels of the *SULTR* genes. In total, four RNA-seq datasets for *C. sativa*, including SRR935368 (root tissue), SRR935362 (leaf tissue), SRR935365 (stem tissue), and SRR935369 (flower tissue) were retrieved from the NCBI gene bank and analyzed. To extract the expression patterns of the *SULTRs* in response to stresses, the RNA-seq datasets related to salt stress (SRR935382), drought stress (SRR935380), cadmium stress (SRR935383), cold stress (SRR935372), and control conditions (SRR935385) were used. For the raw data analysis, we used FastQC software (version 0.11.6) (http://www.bioinformatics.babraham.ac.uk/projects/fastqc/, accessed: 20 September 2022) to check the quality of the data and HISAT [66] to map the sequences. The FPKM (fragments per kilobase of exon model per million mapped reads) metric was used to evaluate the transcription levels of each *SULTR* gene in *C. sativa*. To illustrate the expression levels of the *SULTRs* in *B. napus*, we utilized RNA-seq data for the rapeseed cultivar ZhongShuang11 (ZS11), which were related to 18 tissues and responses to biotic and abiotic stresses, from the Brassica Expression Database [67]. The expression patterns of the target genes were extracted based on their FPKM values. Finally, heatmaps were constructed using the log2 transformed method in TBtools software (version 0.665) [68].

### 4.6. Prediction of 3D Structures, Modeling, Binding Sites, and Phosphorylation

In this study, five proteins from each species (*C. sativa* and *B. napus*) were selected, based on the phylogenetic tree. Additionally, the three-dimensional structures of 10 SULTRs were predicted using the Phyre2 server [69]. In the next step, the predicted structures were checked using a Ramachandran plot analysis [70]. The binding sites of each model were highlighted on the predicted structures. The NetPhos 3.1 server [71], with a potential value of more than 0.90, was used to predict the phosphorylation sites of the SULTRs in *C. sativa* and *B. napus*. 

### 4.7. Expression Patterns of SULTR Genes in C. sativa under Salinity Stress

Sterilized camelina seeds were planted at a depth of 2 cm in pots containing peat moss and were kept under the conditions of 16 h of light and a temperature of 25 °C with irrigation every three days. Then, the five-week-old seedlings were treated with salt (200 mM of NaCl) via irrigation, which was repeated after 24 h. After the salt treatment, leaves were collected at different time points (after 6, 24, and 72 h). The total RNA samples were extracted using an RNX kit (Sinaclon, Iran) and the cDNA was synthesized using a reverse transcriptase kit (Roche, Germany), according to manufacturer protocols. In the present study, five members of the SULTR family were selected for real-time PCR analysis. The genes were selected based on the phylogeny analysis. In addition, *actin-2* (Csa15g026420) was used as a reference gene to normalize the expression data. Specific primers were designed using the Primer3 online software (version 4.1.0) [72], based on the coding sequences of the selected SULTR genes (Table S3). The expression patterns of the SULTR genes were analyzed using a Maxima SYBR Green/ROX qPCR Master Mix kit (Thermo Fisher, France) and the ABI Step One, according to manufacturer protocols. The expression levels of each SULTR gene were calculated using the delta Ct method [73], using three biological replicates.

## 5. Conclusions

In this study, we identified and characterized 36 and 45 putative *SULTR* genes in two important oilseed crops, *Camelina sativa* and *Brassica napus*, respectively. We found that the first duplication event occurred in the *SULTR* genes of *C. sativa* and that members of this family showed diverse structures and functions. Additionally, several *SULTR* genes in *C. sativa* were uniquely developed under evolutionary processes. SULTR 3 was identified as the class of sulfate transporter family genes with the highest diversity. Overall, our results revealed new insights into the structures and functions of SULTRs in oilseed crops. However, further functional studies are needed to evaluate the roles of SULTRs in development and growth processes, as well as in responses to stimuli. Also, investigation of upstream key proteins/enzymes that affect the activity of SULTRs, can reveal the pathways linked to SULTR.

## Figures and Tables

**Table 1:** Summary of SULTRs properties in *Camelina sativa* and *Brassica napus*. Full details of SULTRs properties are provided in Table S1.

Attributes	*C. sativa*	*B. napus*
CDS length (bp)	801–3428	878–3428
Protein length (aa)	266–829	264–758
Exon number	4–20	4–19
pI	7.41–9.93	7.11–10.71
MW (KDa)	29.07–91.99	28.94–83.86
GRAVY	0.271–0.624	0.108–0.621
Instability index	83% stable	73% stable

## Data Availability

Not applicable.

## Supplementary Materials

The following are available online at https://www.mdpi.com/article/10.3390/plants12030628/s1, Table S1: A list of the identified SULTRs and their characteristics in Camelina sativa and Brassica napus, Table S2: The predicted Ka/Ks values in the duplicated gene pairs from the sulfate transporter family in the Camelina sativa and Brassica napus genomes, Table S3: A list of the primers for the camelina SULTR genes that were used in our real-time PCR, Figure S1: The logos of 10 conserved motifs in the sulfate transporter family proteins in Camelina sativa and Brassica napus, Figure S2: A synteny analysis of the SULTR genes in the (a) Camelina sativa and (b) Brassica napus genomes, Figure S3: The distribution of cis-regulatory elements in the SULTR promoter site of Camelina sativa, Figure S4: The distribution of cis-regulatory elements in the SULTR promoter site of Brassica napus.
