# Supplementary figures and images for: Genome-Wide Characterization of the Sulfate Transporter Gene Family in Oilseed Crops: Camelina sativa and Brassica napus
Source: Plants (Basel). 2023 Jan 31;12(3):628. doi: 10.3390/plants12030628 (PMC9919929; doi:10.3390/plants12030628)

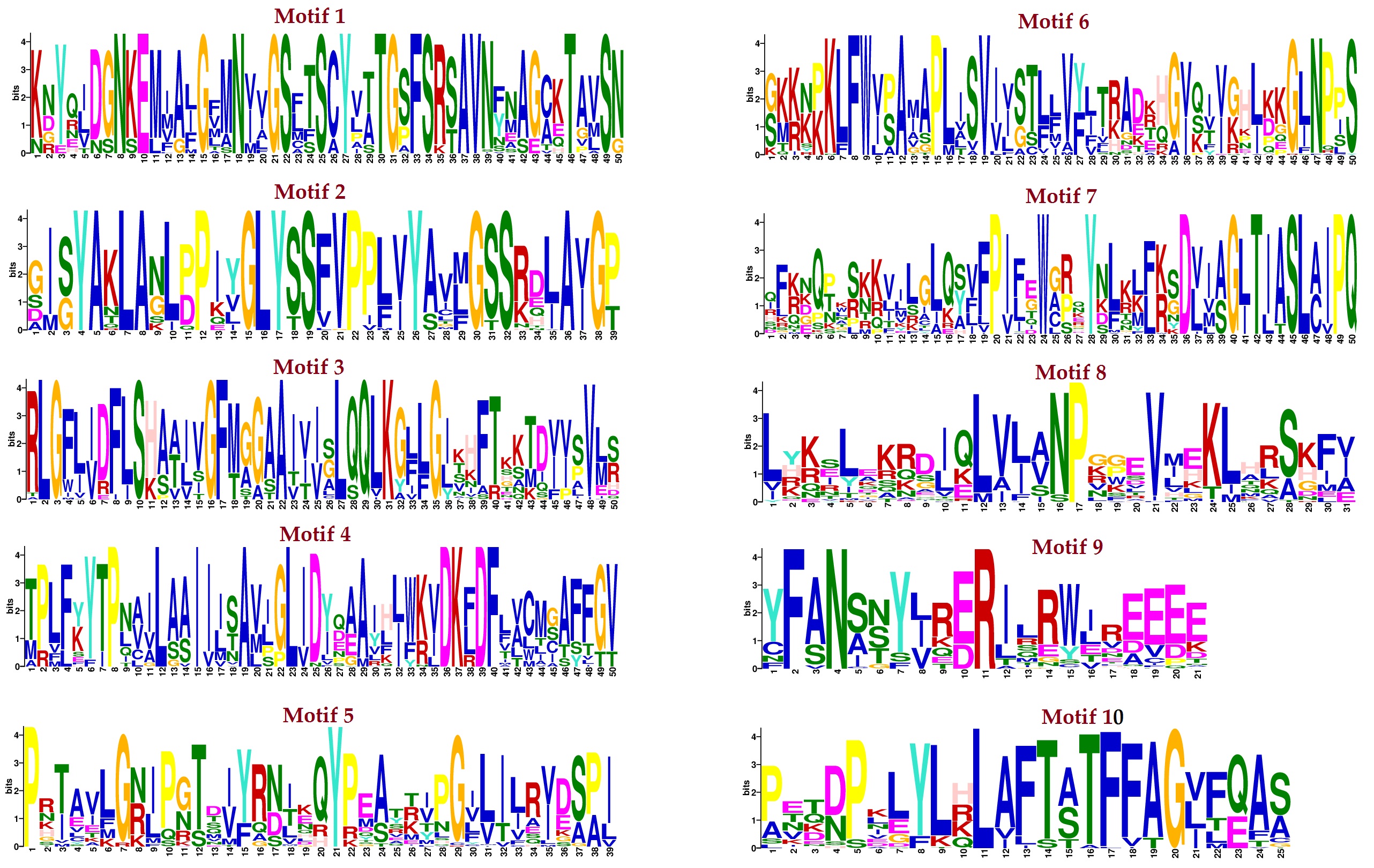

Supplement: Supplementary file 1 [file plants-12-00628-s001.zip › Supp. data/Fig. S1.jpg]

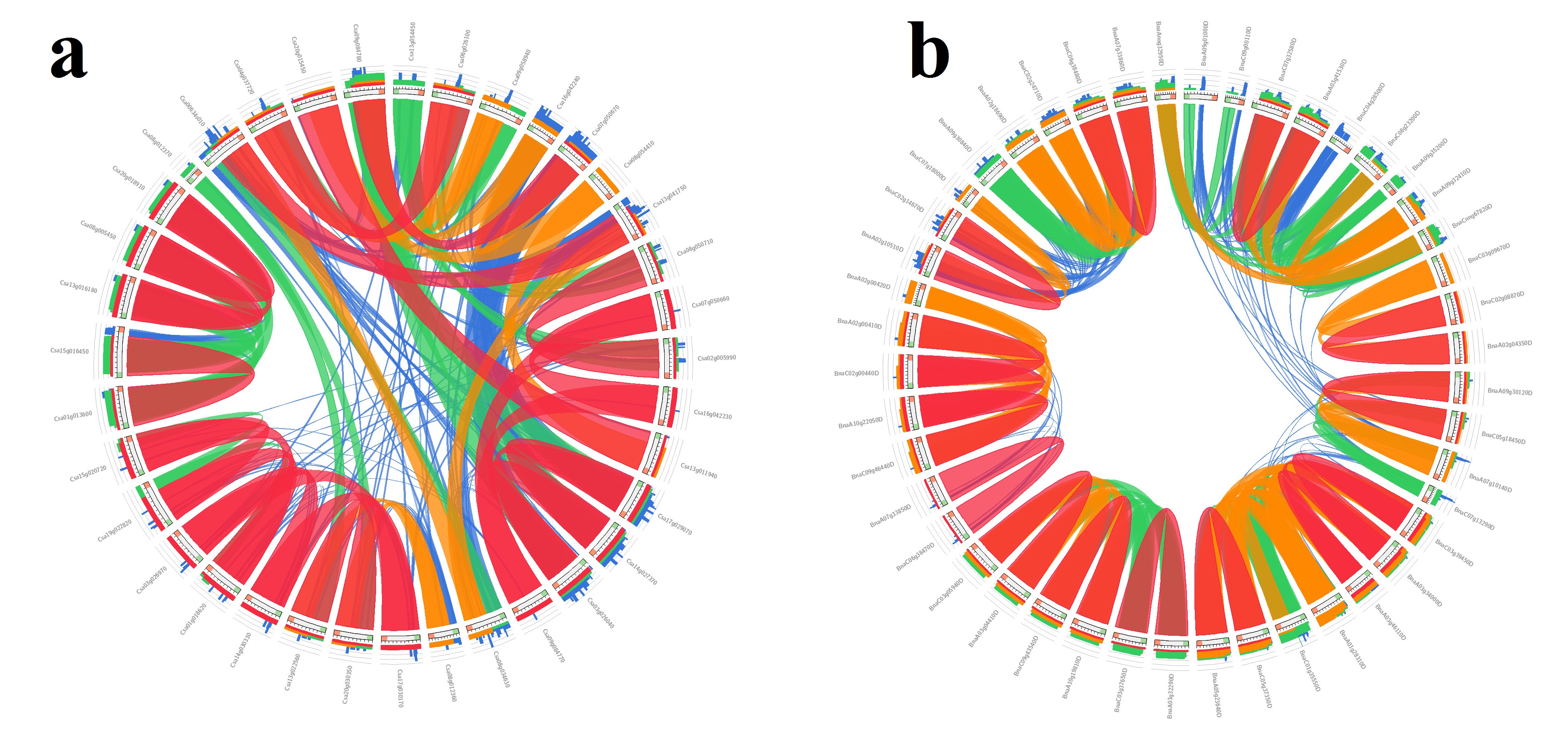

Supplement: Supplementary file 1 [file plants-12-00628-s001.zip › Supp. data/Fig. S2.jpg]

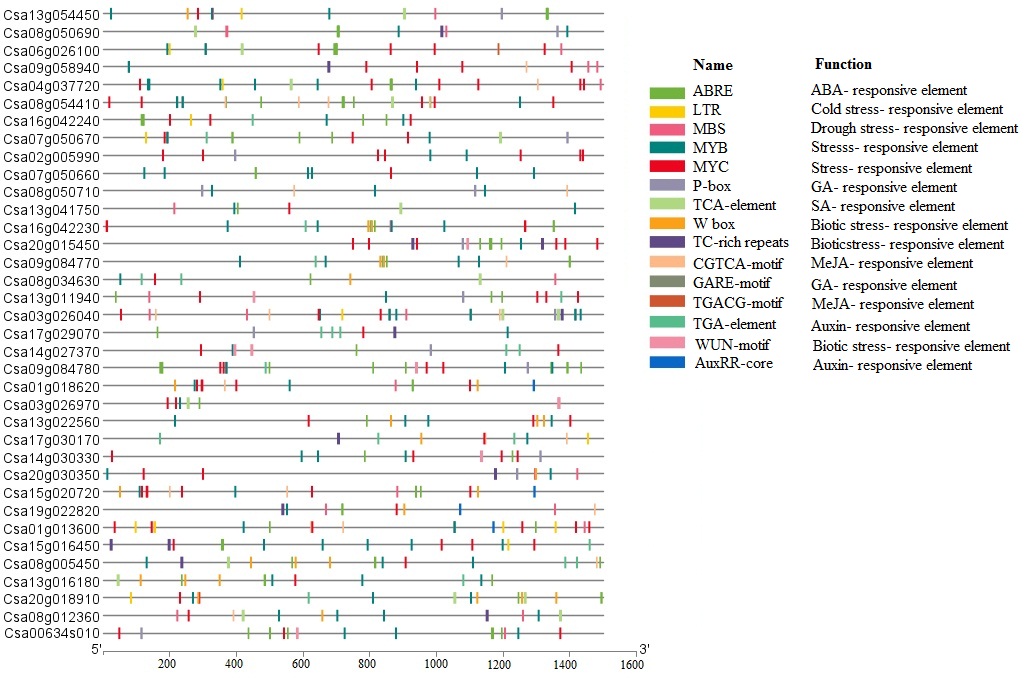

Supplement: Supplementary file 1 [file plants-12-00628-s001.zip › Supp. data/Fig. S3.jpg]

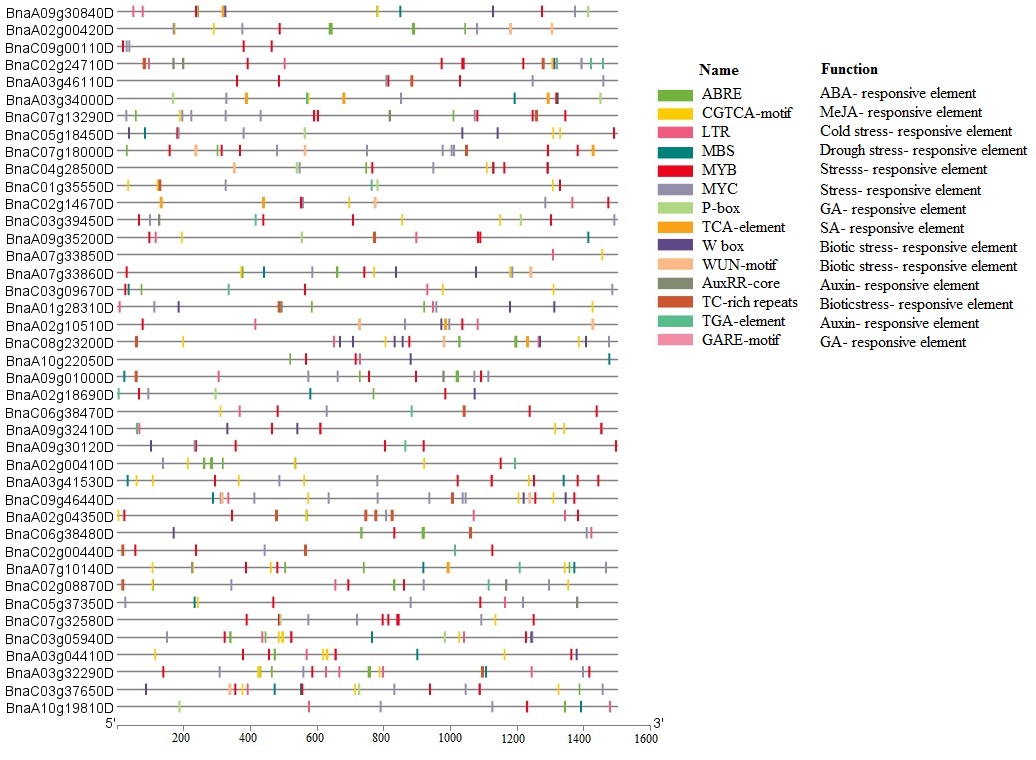

Supplement: Supplementary file 1 [file plants-12-00628-s001.zip › Supp. data/Fig. S4.jpg]
